# Integrated long-read transcriptomic profiling of peripheral blood from ankylosing spondylitis patients identifies regulatory shifts and core genes associated with programmed cell death

**DOI:** 10.3389/fimmu.2026.1640271

**Published:** 2026-03-06

**Authors:** Xue Cao, Panlong Li, Haoran Peng, Qiao Chen, Lipu Shi, Dong Chen, Tianshu Chu, Yanwei Cheng

**Affiliations:** 1 Department of Rheumatology and Immunology, Henan Provincial People’s Hospital, People’s Hospital of Zhengzhou University, People’s Hospital of Henan University, Zhengzhou, China; 2 Department of Emergency, Henan Provincial People’s Hospital, People’s Hospital of Zhengzhou University, People’s Hospital of Henan University, Zhengzhou, China; 3 Nursing Department, Air Force Medical Center, PLA, Beijing, China; 4 Wuhan Ruixing Biotechnology Co., Ltd., Wuhan, China

**Keywords:** alternative splicing, ankylosing spondylitis, isoform switching, long-read sequencing, programmed cell death

## Abstract

Ankylosing spondylitis (AS) is a chronic immune-mediated arthritis marked by persistent inflammation and progressive structural damage. Although dysregulation of programmed cell death (PCD) is increasingly recognized in AS pathogenesis, the full spectrum of transcript-level regulation remains unclear. Here, we employed Oxford Nanopore Technologies (ONT) long-read RNA sequencing to comprehensively profile peripheral blood transcriptomes from six AS patients and six matched healthy controls. Our analysis identified 1,088 differentially expressed genes (DEGs) and 1,812 differentially expressed transcripts (DETs), with upregulated transcripts enriched in apoptosis, autophagy, and transcriptional regulation. We further detected 50 transcripts with significant differential usage and 304 alternative splicing events affecting immune- and PCD-related genes, including *FCGR2B*, *TLR2*, and *STAT5B*. Integrative multilayered analysis revealed 26 core genes, such as *NAMPT*, *GATA2*, and *DDIT3*, showing consistent dysregulation at gene, isoform, and splicing levels, highlighting convergent regulatory networks underlying immune imbalance and cell death in AS. These findings provide the first isoform-resolved transcriptomic landscape of PCD regulation in AS, which unveils extensive regulatory complexity and nominates a set of core genes for future mechanistic and therapeutic exploration.

## Introduction

Ankylosing spondylitis (AS) is a chronic immune-mediated arthritis primarily affecting the sacroiliac joints and axial skeleton, often resulting in persistent pain, structural damage, and functional impairment (1). The global prevalence of AS varies by population, with higher rates observed in individuals carrying human leukocyte antigen B27 (HLA-B27), and a significant disease burden remains due to early onset and lifelong morbidity (2). Despite the advent of biologic therapies, including tumor necrosis factor-alpha (TNF-α) and interleukin-17A (IL-17A) inhibitors, a considerable proportion of patients still experience suboptimal responses or relapse (3, 4). This underscores the need to better understand upstream mechanisms potentially contributing to AS pathogenesis, such as programmed cell death (PCD).

Accumulating evidence implicates dysregulation of programmed cell death (PCD) pathways, including apoptosis, autophagy, necroptosis, and pyroptosis, as central to the pathogenesis and immune dysregulation of AS (5–9). However, transcriptomic investigations in AS currently have predominantly relied on gene-level analyses with short-read sequencing platforms. Such approaches are unable to powerfully resolve isoform diversity or detect alternative splicing events, which represent essential regulatory layers modulating PCD pathways and immune cell fate (10–12). For example, alternative splicing of FAS (CD95) can yield both pro-apoptotic and dominant-negative isoforms, fundamentally altering cellular outcomes (13), yet the scope and impact of such regulatory plasticity in AS remain undefined.

Recent advances in long-read sequencing, such as those offered by Oxford Nanopore Technologies (ONT), now enable accurate profiling of full-length transcripts, thereby capturing isoform usage and splicing complexity with unprecedented resolution (14). These platforms have already revealed novel regulatory shifts in various immune-mediated diseases (15, 16). We hypothesize that AS pathogenesis involves multi-layered transcriptomic disturbances, including differential gene expression, alternative splicing, and isoform switching, that converge to disrupt PCD networks. Integrative analysis of these transcriptomic layers is likely to identify novel molecular targets and mechanistic insights.

In this study, we apply ONT-based long-read RNA sequencing to systematically profile peripheral blood transcriptomes from AS patients and matched healthy controls (HC). By combining analyses of gene expression, isoform usage, and alternative splicing, we generate the first comprehensive landscape of transcriptomic regulation of PCD in AS and identify core genes operating across both transcriptional and post-transcriptional regulatory layers.

## Methods

### Sample collection

Whole blood samples were obtained from 6 patients with AS (3 males and 3 females) who met the modified New York classification criteria, had comparable disease duration and inflammatory levels, and a Bath Ankylosing Spondylitis Disease Activity Index (BASDAI) score ≥ 4. Age- and sex-matched healthy controls (HC) (n = 6) were included. All AS patients had no history of other autoimmune or systemic diseases such as rheumatoid arthritis, systemic lupus erythematosus, inflammatory bowel disease, psoriatic arthritis, psoriasis, or major cardiovascular, hepatic, renal, or hematologic disorders. For all subjects, clinical data including age, sex, disease duration, use of biologics, BASDAI, Bath Ankylosing Spondylitis Functional Index (BASFI), C-reactive protein (CRP), and HLA-B27 status were collected. Detailed participant characteristics are summarized in **Table 1**. Approximately 3~4 mL of whole blood was collected from each individual, and total RNA was extracted for subsequent long-read RNA sequencing using the ONT platform.

**Table 1:** Clinical characteristics of AS patients and healthy controls.

Characteristic	AS	HC	p value
Number	6	6	
Age, years, mean (SD)	32.5 (2.8)	31.7 (3.1)	0.68
Male, n (%)	3 (50%)	3 (50%)	> 0.99
BASDAI, mean (SD)	5.2 (0.6)	—	—
BASFI, mean (SD)	4.3 (0.5)	—	—
CRP (mg/L), mean (SD)	13.2 (2.9)	2.1 (0.4)	< 0.001
HLA-B27, n (%)	6 (100%)	0 (0%)	< 0.0001
Comorbidities	None	None	—

### RNA extraction, library construction, and ONT sequencing

Total RNA was extracted from whole blood using the Total RNA Extraction Kit (DP431, TIANGEN, China), and its purity and concentration were assessed by measuring the absorbance ratio at 260/280 nm using an N50 Touch spectrophotometer (IMPLEN, Germany). RNA integrity was verified by 1% agarose gel electrophoresis. For each sample, 500 ng of total RNA was adjusted to 9 μL with nuclease-free water and mixed with 1 μL of 10 μM VNP primer and 1 μL of 10 mM dNTPs. The mixture was incubated at 65 °C for 5 minutes and then immediately snap-cooled. Strand-switching buffer containing 5 × RT buffer, RNaseOUT, nuclease-free water, and a 10 μM strand-switching primer was added, followed by incubation at 42 °C for 2 minutes. Subsequently, 1 μL of Maxima H Minus Reverse Transcriptase was added, and the reaction proceeded at 42 °C for 90 minutes, followed by heat inactivation at 85 °C for 5 minutes and cooling to 4 °C. For cDNA amplification, 20 μL of first-strand product was combined with 2 μL each of PR1 and PR2 primers, 25 μL of LongAmp Taq 2 × Master Mix, and 1 μL of water. The PCR conditions were: 94 °C for 3 minutes; 12 cycles of 94 °C for 15 seconds, 50 °C for 15 seconds, and 65 °C for 2 minutes; and a final extension at 65 °C for 10 minutes. Barcoding was performed using the ONT Native Barcoding Expansion Kit (SQK-NBD114.96) according to the manufacturer’s protocol. Barcoded DNA was purified using 1 × AMPure XP beads, eluted in 20 μL of nuclease-free water, and quantified using a Qubit fluorometer. Finally, 300 fmol of adapter-ligated library was loaded onto a PromethION R10.4.4 flow cell and sequenced on the ONT platform.

### Long-read sequencing data processing and transcript identification

Long-read RNA sequencing was performed on AS and HC samples using ONT. Raw off-machine data were obtained in POD5 format containing signal-level information. Basecalling was carried out using Dorado (v0.8.2), which converted POD5 files into FASTQ sequences with associated quality scores. Full-length reads were identified and trimmed using Pychopper (v2.7.9), and low-quality reads (mean Q score <10 or length <50 bp) were filtered with Chopper (v0.7.0). Clean reads were aligned to the Ensembl GRCh38_v45 reference genome using Minimap2 (v2.27-r1193) (17) in spliced alignment mode with parameters -ax splice -uf -k14. Isoform-level transcript detection and correction were performed with the Pinfish pipeline, and the resulting polished transcripts were output in GTF format. To create a comprehensive, non-redundant transcriptome across all samples, individual transcript assemblies were merged using StringTie (v2.1.6) (18). Annotation comparison against reference transcripts was conducted using GffCompare (v0.12.6) with parameters -G and -R, allowing classification of novel transcripts and genes (19). Transcript class codes [u, x, i, j, o] were considered novel, while [=, c] indicated known isoforms. All transcripts were merged and retained for downstream quantification and analysis.

### Expression quantification

To quantify expression at both transcript and gene levels, we used Salmon (v1.7.0) (20) in quasi-mapping mode. Transcripts per million (TPM) values were calculated for each transcript and gene across all samples to provide normalized abundance estimates for downstream differential expression analysis.

### Differential expression and transcript usage analysis

Differential expression analysis at both gene and transcript levels was conducted using DESeq2 (v1.30.1) (21), applying thresholds of fold change ≥ 2 and false discovery rate (FDR) ≤ 0.05 to identify differentially expressed genes (DEGs) and differentially expressed transcripts (DETs). Differential transcript usage (DTU) analysis was performed using DEXSeq (22), as implemented in the IsoformSwitchAnalyzeR package (v2.2.0) (23). Transcript annotations generated by GffCompare (v0.12.6) were used as input. For each isoform, the difference in isoform fraction (dIF) between AS and HC groups and its FDR-adjusted p-value were calculated. Isoforms with adjusted p < 0.05 were considered to exhibit significant DTU regardless of dIF value. At the gene level, the lowest adjusted p-value among isoforms was used to assign gene-level significance, and genes with at least one significantly altered isoform were defined as DTU-positive.

### Pathway enrichment analysis

Gene Ontology (GO) terms and Kyoto Encyclopedia of Genes and Genomes (KEGG) pathways (24) were annotated and enriched using KOBAS (v2.0). Statistical significance was assessed by hypergeometric testing, and multiple testing correction was performed using the Benjamini-Hochberg procedure to control the false discovery rate (FDR).

### Differential alternative splicing analysis

Alternative splicing events were identified and quantified using SUPPA2 (v2.3) (25). Splicing levels were measured as percent spliced-in (PSI) values for each event. Differential splicing between AS and HC groups was assessed by calculating the difference in PSI (ΔPSI) and performing independent t-tests. AS events with ΔPSI ≥ 0.05 and p-value ≤ 0.05 were considered significantly differentially spliced and retained for further analysis.

### Programmed cell death analysis

A curated list of genes involved in 12 distinct types of PCD was obtained from **Supplementary Table 1**. These PCD-related genes were cross-referenced with the differential expression, transcript usage, and splicing datasets to identify potential regulatory patterns in AS.

### RNA-seq analysis

RNA-seq datasets (GSE205812 and GSE181364) were downloaded from GEO. The raw reads were trimmed of low-quality bases using a FASTX-Toolkit (v.0.0.13; http://hannonlab.cshl.edu/fastx_toolkit/). Then the clean reads were evaluated using FastQC (http://www.bioinformatics.babraham.ac.uk/projects/fastqc).

The retrieved clean reads were aligned onto the human genome version GRCh38_v45 using HISAT2(v.2.2.1). Uniquely mapped reads were selected for further analysis, and the number of reads located on each gene was calculated. The expression levels of genes were evaluated using FPKM (fragments per kilobase of exon per million fragments mapped). DEseq2 (v.1.30.1) software was used to perform differential gene expression analysis using the reads count file (21). DEseq2 was also used to analyze the differential expression between two or more samples and thus determine whether a gene was differentially expressed by calculating the fold change (FC) and false discovery rate (FDR), FC ≥ 2 or ≤ 0.5, FDR ≤ 0.05.

### Microarray data analysis

The gene expression profiles of microarray datasets GSE25101and GSE73754 were downloaded from GEO. Differential gene expression was performed using limma (26).

### Identification of core AS-related genes

We defined genes showing AS-regulated expression at both gene and transcript levels, as well as AS-regulated alternative splicing as core AS pathogenesis genes. The differential expression of some of these genes was further validated in public datasets.

### Statistical analysis

All statistical analyses and visualizations, including pattern diagrams and stacked bar charts, were performed in R (v4.2.3) using RStudio. Data are presented as mean ± standard error of the mean (SEM). Comparisons between two groups were conducted using Student’s t-test, with p-values < 0.05 considered statistically significant unless otherwise specified.

## Results

### Transcriptome landscape of AS revealed by long-read sequencing

**Figure 1:**
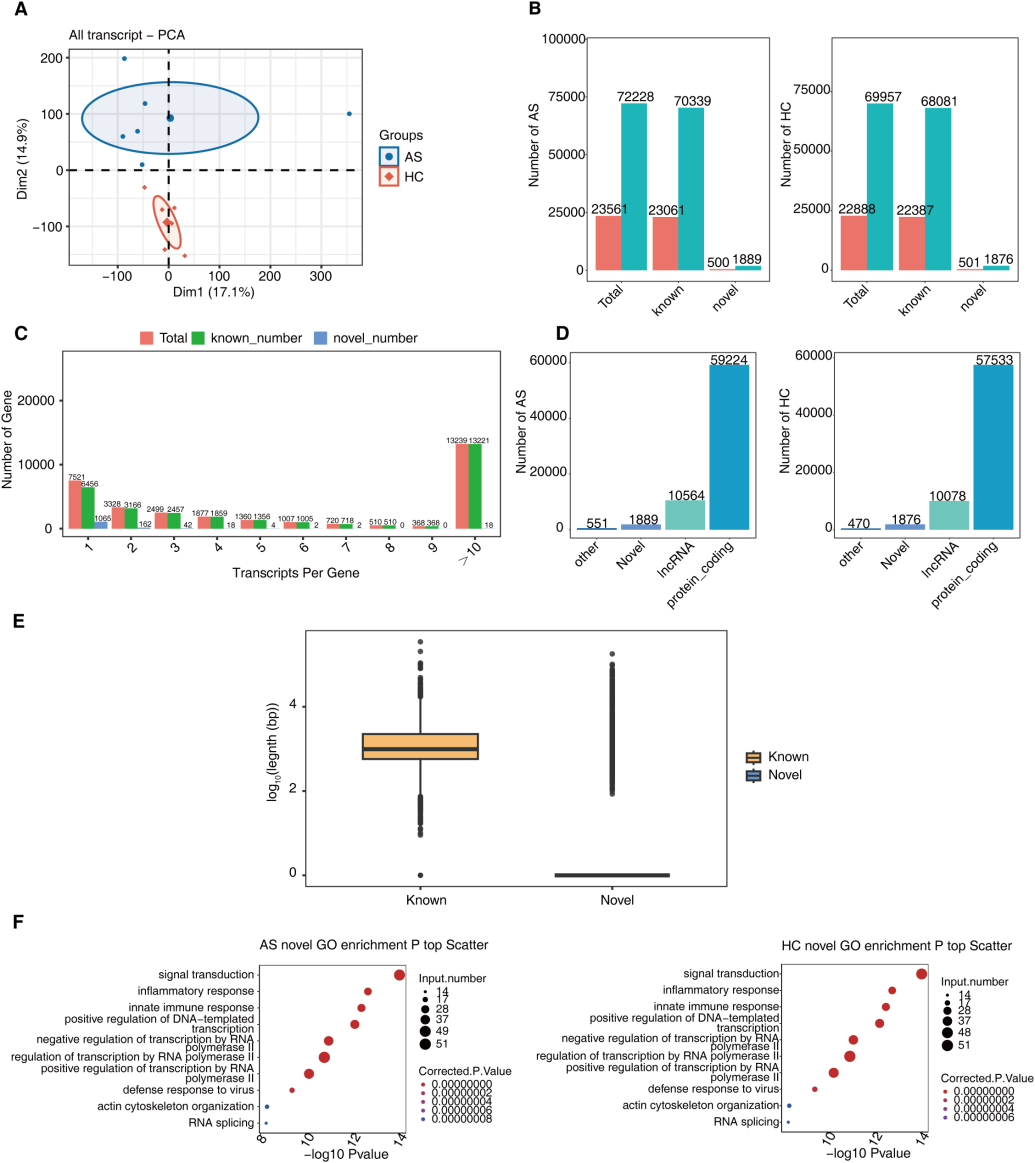
Long-read sequencing reveals transcript expression. **(A)** Principal component analysis (PCA) based on TPM value of all transcripts. The ellipse for each group is the confidence ellipse. **(B)** Bar plots showing the number of transcripts and genes identified from the disease (AS, left panel) and healthy control (HC, right panel) groups. **(C)** The total number of transcriptome isoforms per gene. **(D)** Bar plots showing the number of different types of quantitative transcripts identified from AS (left) and HC (right) groups. **(E)** The box plot showing the length comparison of known and novel, and the ordinate is the length of the transcript taken as log10. **(F)** Scatter plots showing the top 10 most enriched GO terms (biological process) by novel transcripts identified from the AS (left) and HC (right) groups.

Long-read sequencing using ONT was performed on blood samples from 6 AS patients and 6 matched health control (HC) to investigate transcriptomic alterations. The data-analysis strategy in this study is illustrated (**Supplementary Figure 1A**). PCA based on transcript-level TPM values clearly separated AS from HC, indicating distinct global expression profiles (**Figure 1A**). A total of 81,940 transcripts corresponding to 25,343 genes were identified, including 1,902 novel transcripts from 543 novel genes, a slightly larger total number of transcripts (both known and novel) was detected in AS samples compared to HC samples (**Figure 1B**). Many genes exhibited high isoform diversity, with a substantial proportion expressing more than 10 isoforms (**Figure 1C**). Among the novel transcripts, GffCompare classification showed that most were assigned to class codes representing novel isoforms (j, i, o), antisense transcripts (x), or intergenic loci (u), suggesting the presence of previously unannotated transcriptional activity (**Supplementary Figures 2A–C**). The majority of quantified transcripts were protein-coding, and novel transcripts were generally shorter than known ones, as shown by the log-transformed length comparisons (**Figures 1D, E**). We performed functional enrichment analysis with genes associated with novel transcripts in AS and HC groups, demonstrating that the significantly enriched GO terms in both groups were similarly related to AS pathogenesis including inflammatory response, innate immune response, and actin cytoskeloton organization (**Figure 1F**). GO terms and KEGG pathways enriched by all transcripts detected in this study were also shown. AS related KEGG pathways included osteoclast differentiation, autophagy, and antigen processing (**Supplementary Figures 2C, D**). These findings highlight the extensive transcriptomic complexity in AS and suggest a functional relevance of novel isoforms in disease-related pathways.

### Differential transcript expression landscape in AS

**Figure 2:**
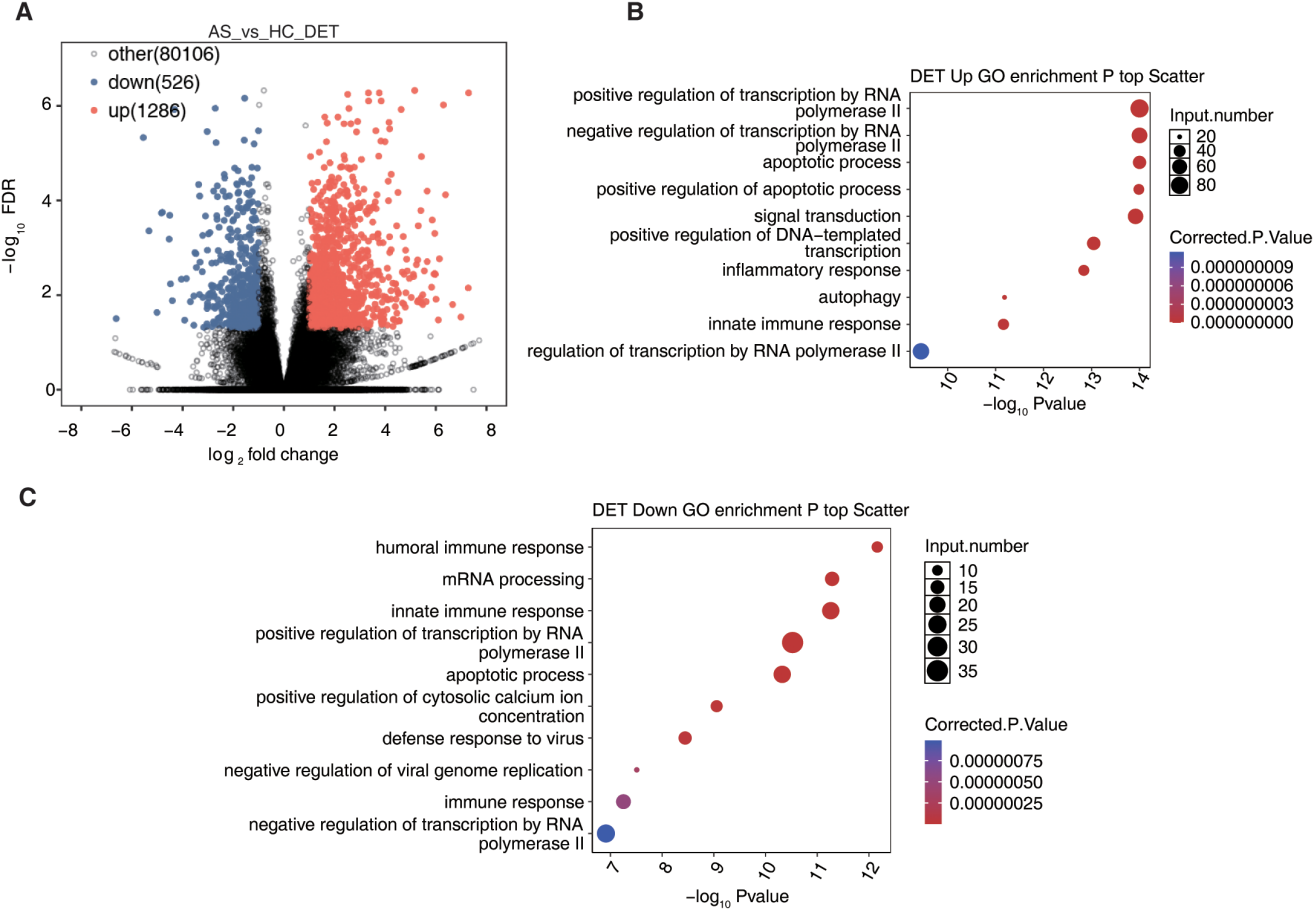
Long-read sequencing reveals the transcript expression landscape in ankylosing spondylitis. **(A)** Volcano plot showing differential transcripts analyzed by DESeq2 (FC ≥ 2 or ≤ 0.5, FDR ≤ 0.05). **(B, C)** The top 10 most enriched GO terms (biological process) were illustrated for genes of up-regulation transcripts and down-regulation transcripts.

We further characterized transcript-level expression differences between AS and HC, identifying 1,812 DETs, including 1,286 upregulated and 526 downregulated transcripts (**Figure 2A**). The broad distribution of DETs underscores extensive isoform-level alterations in AS. GO enrichment analysis revealed that upregulated DETs were significantly associated with transcriptional regulation via RNA polymerase II, apoptotic signaling, inflammation, autophagy, and innate immune activation (**Figure 2B**). In contrast, downregulated DETs were enriched in biological processes including humoral immune response, mRNA splicing and processing, antiviral defense, and regulation of cytoplasmic calcium ion levels (**Figure 2C**). These findings suggest that AS involves coordinated remodeling at the transcript level, characterized by activation of pro-inflammatory and cell death pathways and suppression of immune regulatory and RNA processing functions.

### Differential gene expression profile in AS

**Figure 3:**
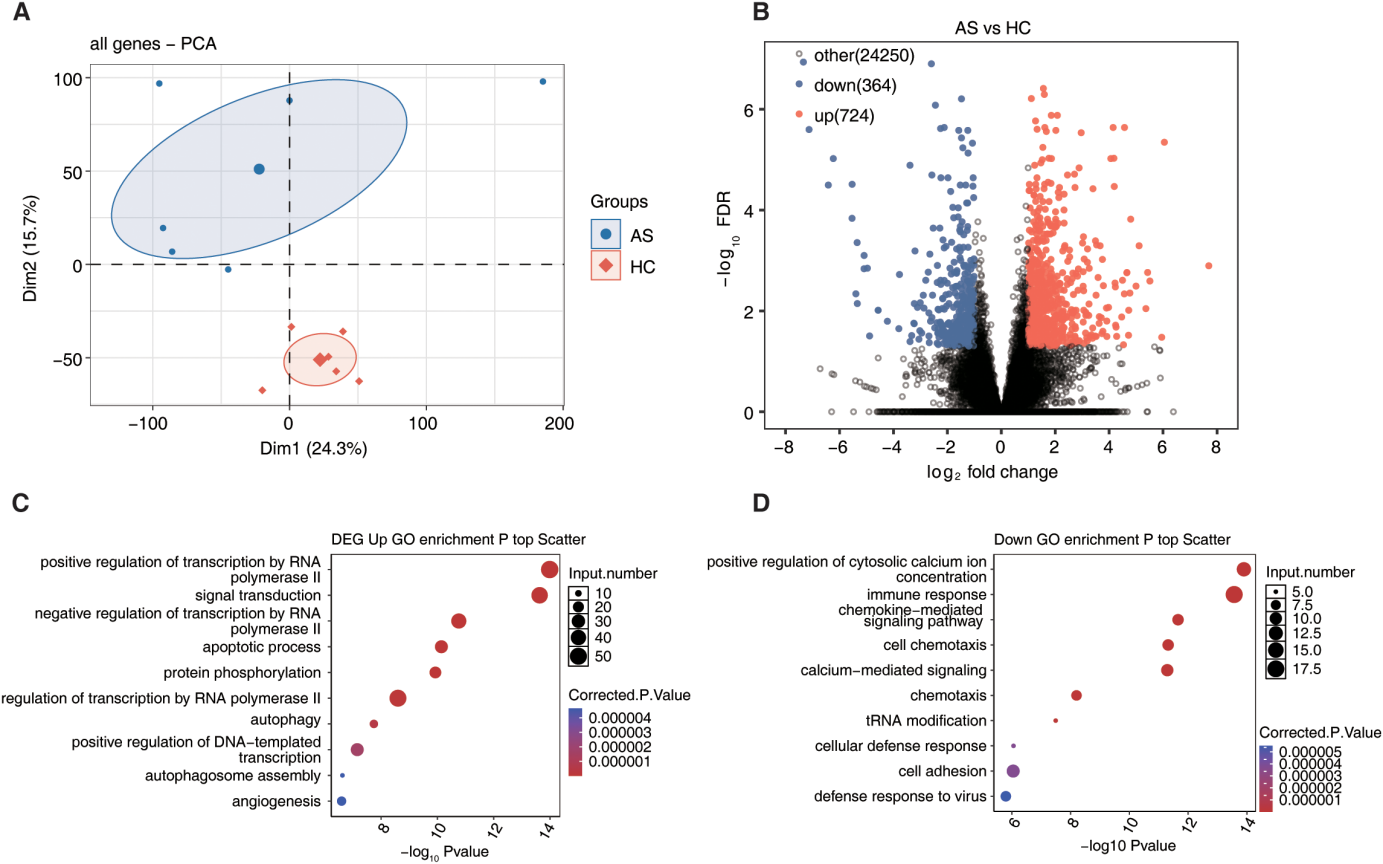
Long-read sequencing reveals the gene expression profile in ankylosing spondylitis. **(A)** Principal component analysis (PCA) based on TPM value of all genes. The ellipse for each group is the confidence ellipse. **(B)** Volcano plot showing differential genes analyzed by DESeq2 (FC ≥ 2 or ≤ 0.5, FDR ≤ 0.05). **(C, D)** The scatter plot shows the most abundant GO biological process results of up-regulation and down-regulation gene.

To investigate gene-level alterations in AS, we performed PCA based on gene-level TPM values, which revealed distinct separation between AS and HC samples, indicating overall differences in gene expression profiles (**Figure 3A**). We subsequently identified 1,088 DEGs between the two groups, including 724 upregulated and 364 downregulated genes, with a notable shift toward upregulation in AS (**Figure 3B**). GO enrichment analysis showed that upregulated DEGs were predominantly involved in transcriptional activation via RNA polymerase II, apoptosis, autophagy, and angiogenesis, suggesting increased cellular activity and tissue remodeling in AS (**Figure 3C**). In contrast, downregulated genes were enriched in chemokine signaling, calcium-mediated pathways, innate immune suppression, and host defense processes (**Figure 3D**). Together, these findings indicate a global shift in gene expression that promotes inflammation, stress response, and immune modulation in AS.

### Differential expression of programmed cell death-related genes and transcripts in AS

**Figure 4:**
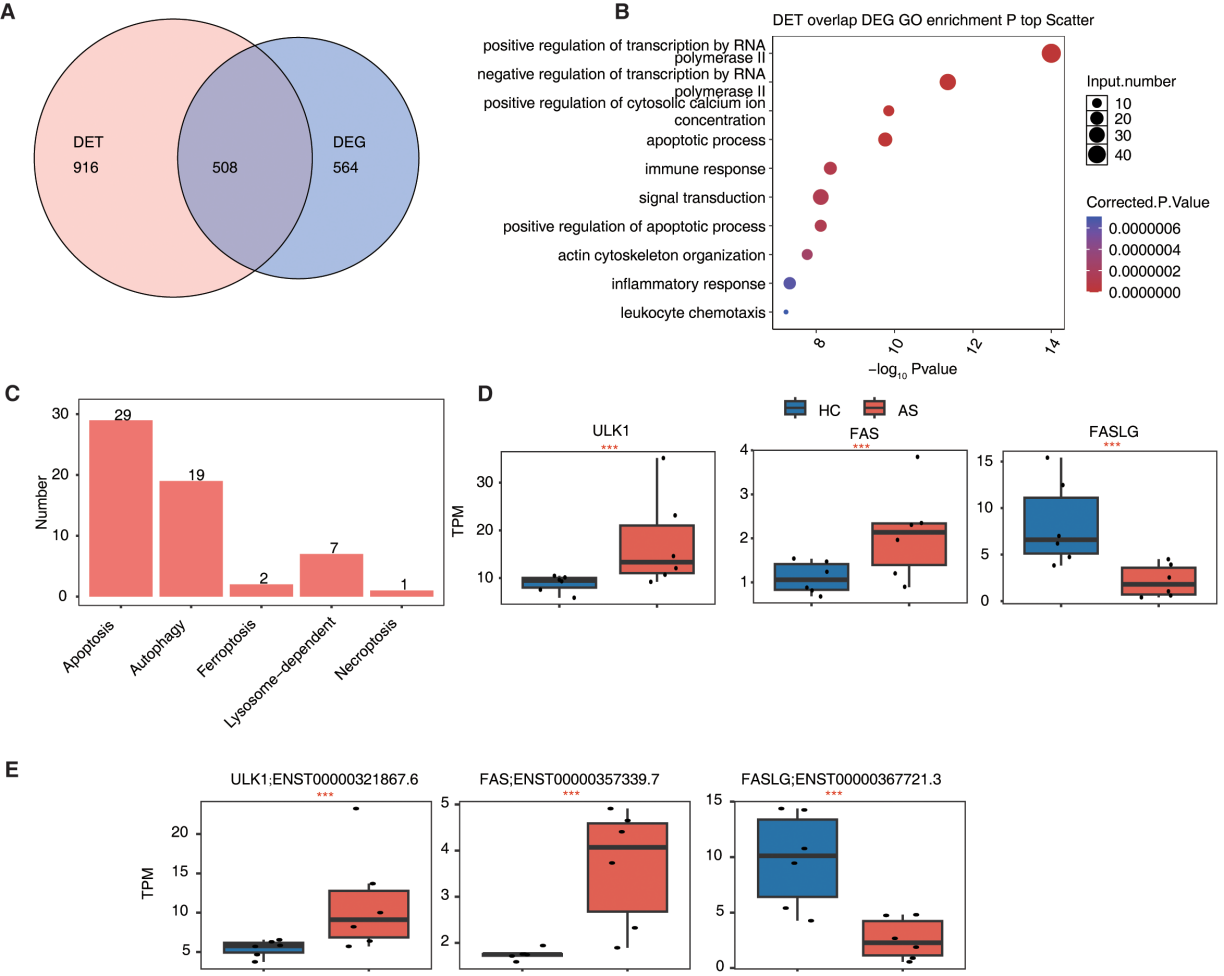
Differential expression of transcripts and genes related to programmed cell death in ankylosing spondylitis. **(A)** The Venn diagram shows the overlap between DET and DEG. **(B)** The scatter plot shows the most abundant GO biological process results of DET overlap DEG. **(C)** The bar plot shows the overlap number of overlap genes related to Programmed Cell deaths (PCD). **(D)** The box plot shows the DEG of TPM between the AS and HC group. ***P value ≤ 0.001. **(E)** The box plot shows the DET of TPM between the AS and the HC group. ***P value ≤ 0.001.

To explore the involvement of PCD pathways in AS, we integrated DETs and DEGs, identifying 508 overlapping genes between the two datasets (**Figure 4A**). GO enrichment analysis of these overlapping genes revealed their significant association with biological processes including apoptosis, calcium signaling regulation, innate and adaptive immune responses, and cytoskeletal organization (**Figure 4B**). To further examine their relevance to PCD, we cross-referenced the 508 overlapping genes with curated databases covering 12 types of cell death. A substantial number were associated with apoptosis (29 genes), autophagy (19 genes), lysosome-dependent cell death (7 genes), ferroptosis (2 genes), and necroptosis (1 gene) (**Figure 4C**), indicating broad transcriptional involvement of PCD-related mechanisms in AS. Among them, *FAS* (Fas cell surface death receptor), *FASLG* (Fas ligand), and *ULK1* (unc-51 like autophagy activating kinase 1), key regulators of apoptosis and autophagy, exhibited consistent and significant differential expression at both gene and transcript levels. Specifically, *FAS* and *ULK1* were upregulated, while *FASLG* was downregulated in AS compared to HC (**Figures 4D, E**). These findings suggest that dysregulated expression of core PCD components may contribute to immune and inflammatory imbalances in AS.

### Differential transcript usage in AS

**Figure 5:**
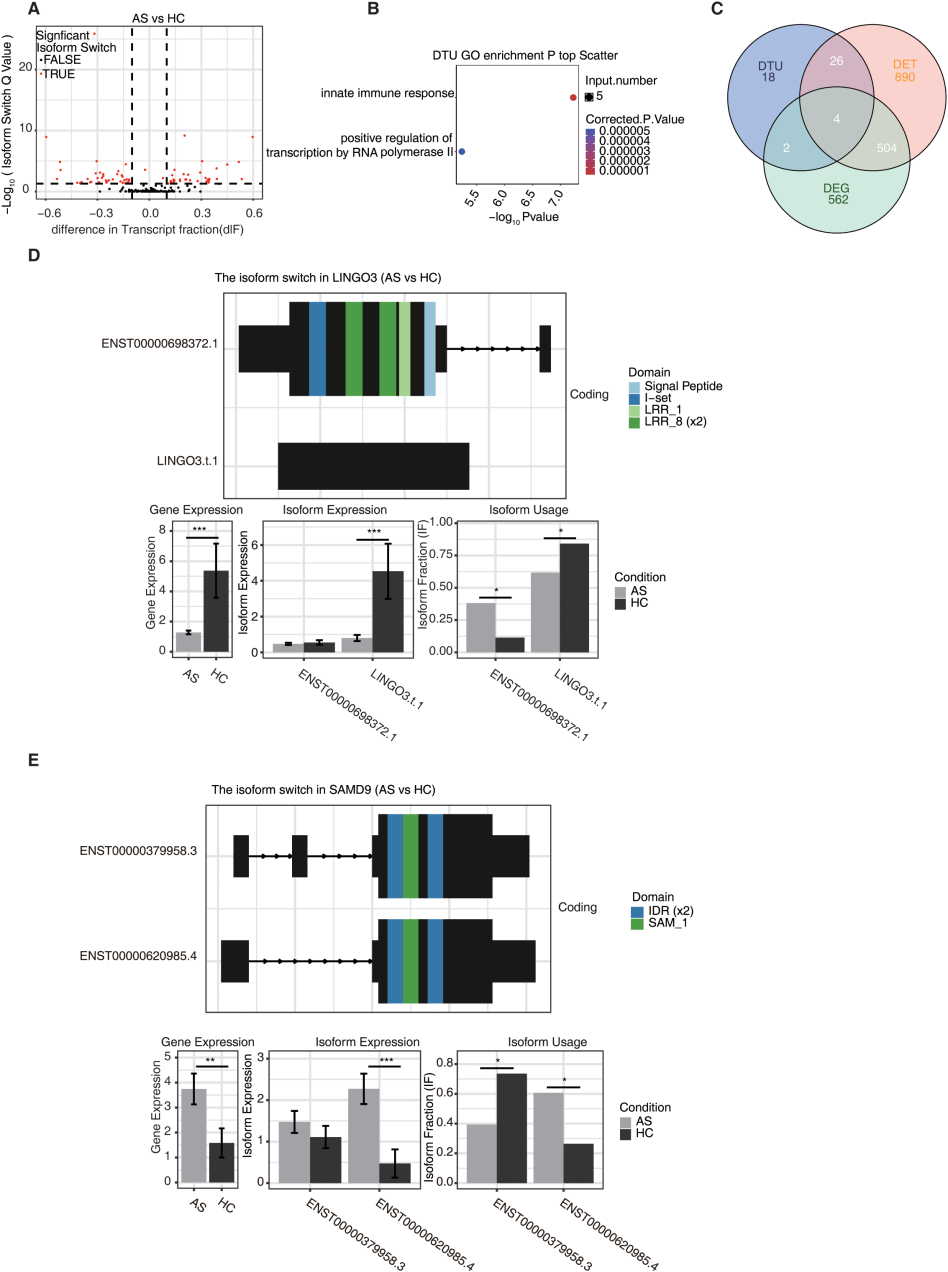
Long-read sequencing reveals the differential transcript usage in ankylosing spondylitis. **(A)** The volcano plot, generated by IsoformSwitchAnalyzeR, depicts transcript switching across the AS and HC groups. The x - axis depicts the difference in transcript fraction (dIF) for a given transcript in the AS versus control. The y - axis represents - log10 (p - value) of the transcript switch, where a higher value indicates a more significant transcript switch. **(B)** The scatter plot shows the most abundant GO biological process results of DTU. **(C)** The Venn diagram shows the overlap between DET and DEG and DTU. **(D, E)** Isoform switching observed within the LINGO3 and SAMD9 is presented in this figure generated by IsoformSwitchAnalyzeR. The upper part shows the isoform switch with colors denoting different functional domains. The lower part has three sub - plots for gene expression, isoform expression, and isoform fraction (IF). Asterisks (*P value ≤ 0.05, **P value ≤ 0.01, ***P value ≤ 0.001) mark significant differences between AS and HC samples, revealing insights into LINGO3’s and SAMD9’s isoform - specific regulation.

To further investigate transcript-level regulatory dynamics in AS, we analyzed DTU between AS and HC using DEXSeq implemented in IsoformSwitchAnalyzeR. A total of 50 transcripts exhibited significant differences in isoform usage, independent of overall gene expression changes (**Figure 5A**). GO enrichment analysis revealed that these DTU events were predominantly associated with innate immune response and regulation of RNA polymerase II transcription (**Figure 5B**). Integration of DTU with DET and DEG datasets identified 4 overlapping genes, including *LINGO3* (leucine rich repeat and Ig domain containing 3) and *SAMD9* (sterile alpha motif domain containing 9) (**Figure 5C**), both of which displayed distinct isoform usage alterations between AS and HC (**Figures 5D, E**). In *LINGO3*, although total gene expression was decreased in AS, the dominant isoform was preferentially used, while the alternative isoform showed reduced usage. In *SAMD9*, gene and isoform expression levels were relatively similar between groups, but the two isoforms exhibited opposite usage patterns between AS and HC, indicating a pronounced switch in isoform preference. These isoform changes were also associated with protein domain differences, suggesting potential functional divergence. Notably, these usage alterations occurred without proportional changes in isoform expression, reinforcing their regulatory specificity. Together, these findings support isoform-level regulation as an additional layer contributing to transcriptomic remodeling in AS.

### Alternative splicing landscape in AS

**Figure 6:**
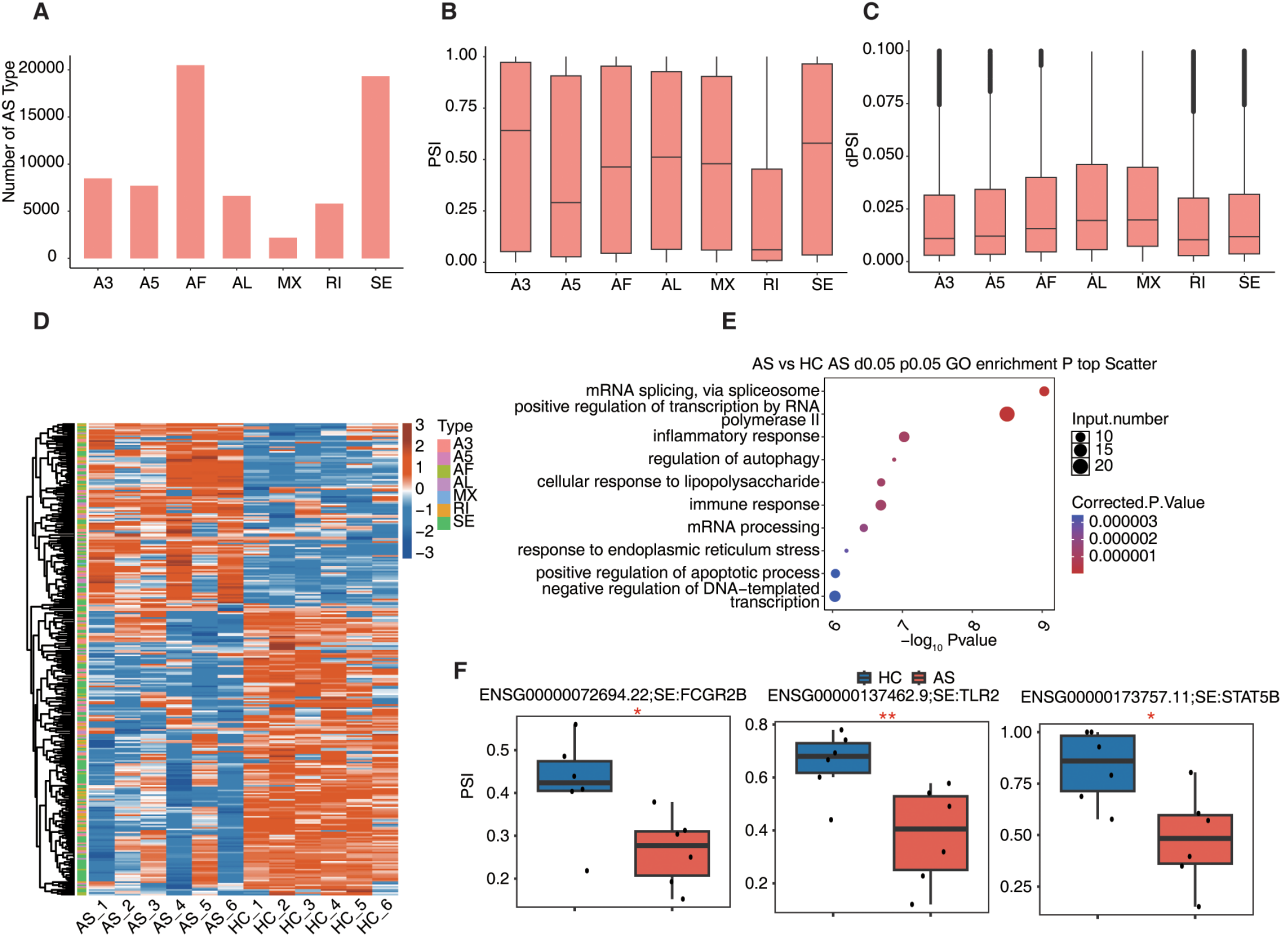
Long-read sequencing reveals alternative splicing in ankylosing spondylitis. **(A)** The bar graph shows all types of alternative splicing identified by Suppa2. **(B)** bar plots show PSI of all alternative splice types identified by Suppa2. **(C)** bar plots show PSI differences for all types of alternative splicing identified by Suppa2. **(D)** Heatmap showing PSI of all types of alternative splicing for each sample identified by Suppa2. **(E)** The scatter plot shows the most abundant GO biological process results of differential AS. **(F)** Boxplots showing the difference in PSI of alternative splicing between AS and HC groups. We compute significance by using the empirical method of Suppa2. *P value ≤ 0.05, **P value ≤ 0.01.

Building on the observed isoform-level changes, we next explored alternative splicing regulation to better understand the mechanisms underlying transcript diversity in AS. A total of 7 types of alternative splicing were identified, including exon skipping (SE), retained introns (RI), mutually exclusive exons (MX), alternative 5′ and 3′ splice site usage (A5, A3), as well as alternative first exons (AF) and alternative last exons (AL), with AF being the most prevalent AS type, followed by SE (**Figure 6A**). Distributions of PSI values and corresponding ΔPSI metrics showed global variability between AS and HC (**Figures 6B, C**). Following statistical filtering, 304 significantly altered splicing events were retained, which clearly separated AS from HC samples in the PSI-based splicing heatmaps (**Figure 6D**). GO enrichment analysis of alternatively spliced genes indicated functional involvement in mRNA splicing, transcriptional regulation, cellular stress responses, autophagy, and apoptotic signaling (**Figure 6E**), suggesting that AS-related splicing alterations may affect key immune and cell death pathways. Representative examples of disease-relevant splicing events included reduced exon inclusion in *FCGR2B* (Fc gamma receptor IIb), *TLR2* (Toll-like receptor 2), and *STAT5B* (Signal transducer and activator of transcription 5B) in AS compared to HC (**Figure 6F**). These splicing changes may alter isoform structure and function, potentially impacting immune regulation and inflammatory signaling in AS.

### Integration of alternative splicing and transcript expression reveals programmed cell death regulation in AS

**Figure 7:**
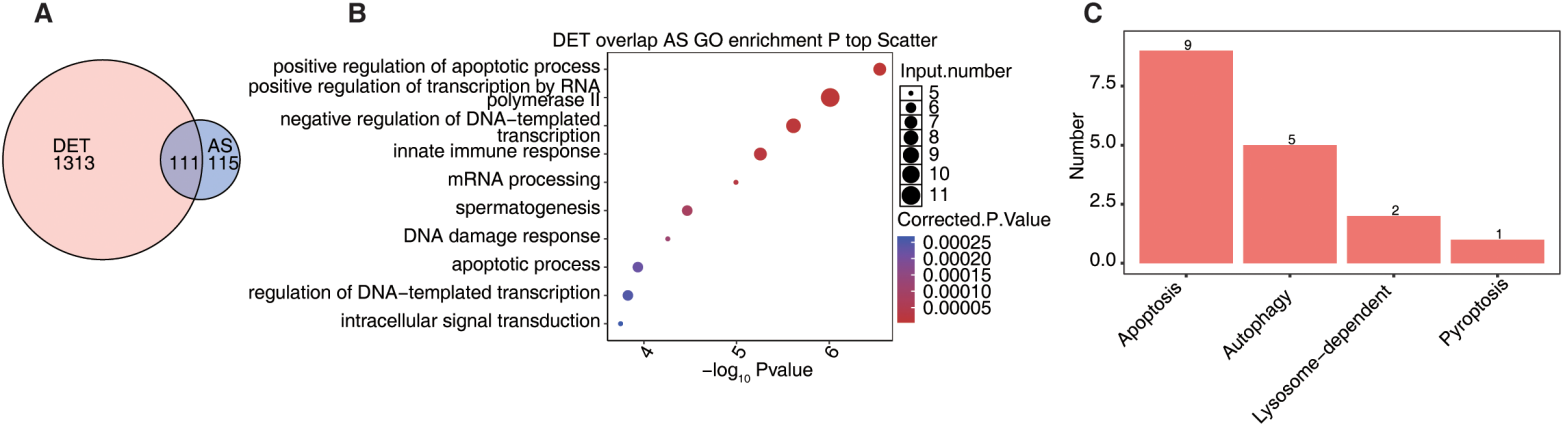
Alternative splicing affects the regulation of transcript expression related to programmed cell death in ankylosing spondylitis. **(A)** The Venn diagram shows the overlap between DET and AS. **(B)** The scatter plot shows the most abundant GO biological process results of DET overlap AS. **(C)** The bar plot shows the overlap number of overlap genes related to Programmed Cell deaths (PCD).

To investigate whether alternative splicing contributes to the regulation of PCD-related transcript expression in AS, we integrated DETs with genes exhibiting significant AS events and identified 111 overlapping genes affected at both the splicing and transcript levels (**Figure 7A**). GO enrichment analysis of these genes revealed strong associations with apoptotic signaling, innate immune responses, mRNA processing, DNA damage repair, and transcriptional regulation (**Figure 7B**), suggesting their functional relevance in AS pathology. Cross-referencing with curated PCD gene sets showed that these dual-regulated genes included 9 related to apoptosis, 5 to autophagy, and others involved in lysosome-dependent and pyroptotic pathways (**Figure 7C**). These findings indicate that alternative splicing may cooperate with transcript-level expression changes to modulate cell death-associated genes, contributing to immune dysregulation and disease progression in AS.

### Multilayer regulatory convergence identifies core genes associated with AS pathogenesis

**Figure 8:**
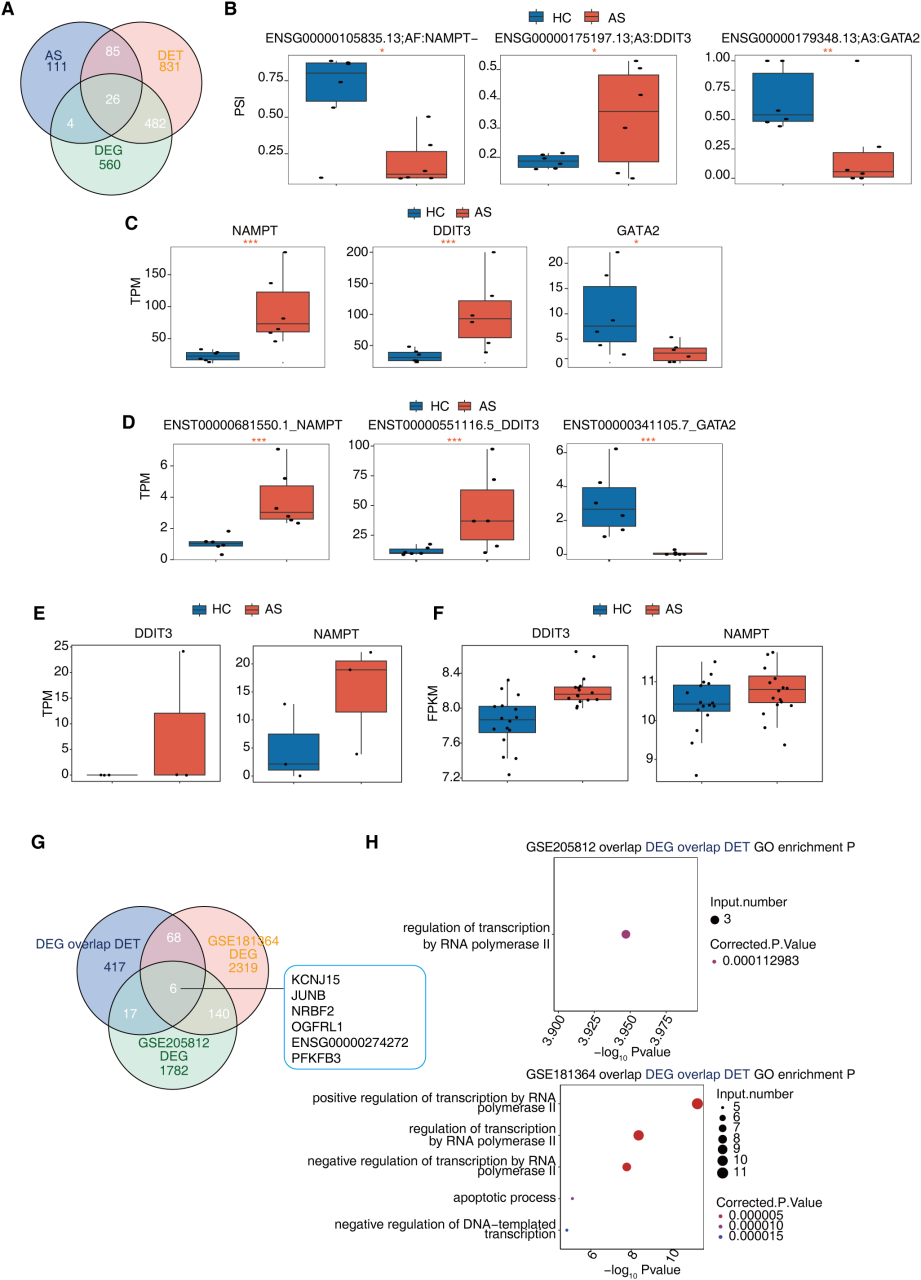
Analysis of genes with transcript expression differences due to alternative in ankylosing spondylitis. **(A)** The Venn diagram shows the overlap between DET, AS and DEG. **(B)** Boxplots showing the difference in PSI of alternative splicing between AS and HC groups. We compute significance by using the empirical method of Suppa2. *P value ≤ 0.05, **P value ≤ 0.01, ***P value ≤ 0.001. **(C)** The box plot shows TPM of representative DEGs between the AS and the HC group. *P value ≤ 0.05, **P value ≤ 0.01, ***P value ≤ 0.001. **(D)** The box plot shows TPM of representative DETs between the AS and the HC group. *P value ≤ 0.05, **P value ≤ 0.01, ***P value ≤ 0.001. **(E)** The box plot shows TPM of *NAMPT* and *DDIT* between the AS and the HC group from RNA-seq dataset GSE205812. **(F)** The box plot shows expression levels of *NAMPT* and *DDIT* between the AS and the HC group from microaaray dataset GSE25101. **(G)** Venn plot of the overlap among the shared DEG/DET identified in this study and DEGs identified from datasets GSE205812 and GSE181364. **(H)** The scatter plot of GO terms of the overlapped DEGs between different datasets shown in **(G)**.

To pinpoint key genes under coordinated transcriptional and post-transcriptional regulation in AS, we performed an integrative analysis combining DEGs, DETs and alternative splicing events. This approach identified 26 genes exhibiting concurrent dysregulation at the levels of gene expression, transcript expression and splicing regulation, highlighting candidates potentially central to AS pathogenesis (**Figure 8A**). Among them, *NAMPT* (Nicotinamide Phosphoribosyltransferase), *GATA2* (GATA Binding Protein 2), and *DDIT3* (DNA Damage Inducible Transcript 3) were notable for their consistent and significant dysregulation across all three regulatory layers. These genes also showed significant differences in PSI values (**Figure 8B**), suggesting altered splicing regulation. Gene-level expression analysis revealed upregulation of *NAMPT* and *DDIT3* and downregulation of *GATA2* in AS (**Figure 8C**), while transcript-level data confirmed isoform-specific expression changes (**Figure 8D**). Functionally, each of these genes is intimately linked to PCD pathways. These results identify a subset of genes dysregulated across multiple transcriptomic layers in AS, many of which are functionally linked to immune regulation and programmed cell death pathways.

### The up-regulated expression of *NAMPT* and *DDIT3* in AS is confirmed by additional datasets

We then downloaded four other available PBMC transcriptomic datasets to investigate the AS-deregulated core gene expression. We found that the expression of both *NAMPT* and *DDIT3* was increased in one RNA-seq dataset (GSE205812) and one microarray dataset (GSE25101), although the increase did not reach statistical significance (**Figures 8E, F**), while the down-regulated expression of *GATA2* was not evident (data not shown). This finding supports the potential importance of *NAMPT* and *DDIT3* in AS pathobiology.

We noted that differential was marginal in microarray datasets on AS (GSE25101and GSE73754), with only a few genes identified as differentially expressed (data not shown). We performed GO analysis of the overlapped genes between the 508 AS-regulated DEGs/DETs identified in this study (**Figure 4A**) and AS-regulated DEGs identified from two previously published RNA-seq datasets (GSE205812 and GSE181364) (**Figure 8G**). These overlapping genes were enriched in transcriptional regulation and the apoptotic process (**Figure 8H**).

## Discussion

Our integrated long-read transcriptomic analysis in AS systematically revealed a multilayered landscape of transcriptomic dysregulation, encompassing differential gene and transcript expression, isoform usage, and alternative splicing, particularly within PCD pathways. This represents the first study in AS to comprehensively dissect transcriptional and post-transcriptional regulatory alterations at isoform resolution, and despite the exploratory nature of our cohort (n=6 per group), it provides a novel, high−resolution molecular map that highlights previously inaccessible regulatory layers in AS. We acknowledge that the sample size, though typical for initial long−read sequencing studies due to cost and analytical complexity, limits statistical power and generalizability. Nevertheless, the consistent separation of AS and HC groups in global expression and splicing profiles, together with stringent statistical thresholds and external validation of key findings (see below), supports the robustness of the reported transcriptomic landscape.

In our cohort, we identified 1,088 DEGs and 1,812 DETs distinguishing AS from HC, with a strong bias towards upregulation. Functional enrichment highlighted apoptosis, autophagy, angiogenesis, and transcriptional regulation among upregulated genes and transcripts, whereas downregulated ones were linked to chemokine signaling and mRNA splicing. This pattern reflects a transcriptional shift toward activation of cell death and cellular stress pathways, together with suppression of certain immune regulatory processes, which may contribute to chronic inflammation and tissue remodeling in AS (27, 28). Importantly, many DETs represented isoform - specific changes not captured by conventional gene - level analysis, highlighting the unique capacity of long-read sequencing to uncover previously hidden layers of transcriptomic complexity in autoimmune disease (29).

A unique advantage of our long-read approach is the identification of 50 transcripts with significant DTU between AS and HC. This highlights a layer of regulatory divergence beyond overall gene expression. For example, we observed pronounced shifts in isoform fractions of *LINGO3* and *SAMD9* in AS. LINGO3 is implicated in mucosal inflammation, whereas SAMD9 is involved in antiviral defense and interferon signaling (30, 31). These isoform changes may influence domain composition and downstream signaling. Previous research in autoimmune diseases has shown that isoform switching can act as a molecular switch in immune activation and tolerance, underscoring the pathogenic relevance of DTU (32). Together, these findings suggest that transcript usage modulation is an important but underappreciated mechanism underlying immune dysregulation in AS.

Integrative analysis across gene, transcript, and splicing layers pinpointed 26 core genes with consistent multi-level dysregulation. Among these, *NAMPT*, *GATA2*, and *DDIT3* emerge as focal regulators within PCD networks. emerged as focal regulators within PCD networks. These genes exhibited convergent dysregulation at expression, isoform, and splicing levels, suggesting that multi−layered regulatory convergence is a hallmark of PCD and immune dysfunction in AS. NAMPT is a key NAD^+^ biosynthetic enzyme involved in energy metabolism, inflammation, and neutrophil extracellular trap formation, and has been targeted in rheumatoid arthritis models (33). GATA2 is essential for hematopoietic stem cell maintenance and immune lineage specification. In addition to its role in natural killer (NK) cell development. GATA2 also regulates transcriptional programs involved in immunological tolerance and leukemogenesis, with mutations linked to familial myelodysplastic syndromes and increased infection susceptibility, and its dysregulation may affect immune−cell development and function in AS (34, 35). DDIT3 (also known as CHOP) is a canonical ER stress sensor promoting apoptosis and ferroptosis under oxidative and metabolic stress (7, 36). The observation that these genes are dysregulated at both expression and splicing levels suggests that multi-layered regulatory convergence is a hallmark of PCD and immune dysfunction in AS.

Notably, the up-regulation of NAMPT and DDIT3 was also observed in two independent peripheral blood transcriptomic datasets (GSE205812, RNA-seq; GSE25101, microarray) (**Figures 8E, F**), reinforcing their potential relevance in AS. Furthermore, comparison of our DEG/DET set with DEGs from two publicly available RNA-seq studies (GSE205812, GSE181364) revealed a significant overlap enriched in apoptotic and transcriptional regulatory processes (**Figures 8G, H**), indicating that PCD-related transcriptional remodeling is a recurrent feature across AS blood transcriptomic studies (37). For instance, JUNB—a shared DEG in our and two prior datasets—has been implicated in AS pathogenesis through single-cell multi-omics studies (38).

Although our data are derived from peripheral blood, a readily accessible but indirect compartment, the observed transcriptomic shifts likely reflect systemic immune dysregulation that may contribute to or mirror processes in affected tissues. Circulating immune cells can serve as both mediators and reporters of inflammation, and shared pathways between blood and synovial/entheseal tissue have been documented in spondyloarthritis (39). Thus, the isoform-level alterations identified here provide a set of testable hypotheses for future investigations in target tissues or appropriate cellular models.

Furthermore, our analysis revealed that diverse PCD mechanisms are transcriptionally intertwined in AS pathogenesis. Prior research has largely focused on necroptosis and autophagy in synovial inflammation, but our data show co-regulation across multiple PCD subtypes, including lysosome-mediated cell death and pyroptosis. Recent studies have shown that these forms of cell death interact dynamically with inflammation, affecting IL-1β release, macrophage polarization, and tissue damage (6, 40, 41). The link between PCD dysregulation and HLA-B27 misfolding pathways may further connect our transcriptomic observations with known molecular mechanisms of AS. Importantly, recent advances in targeting cell death regulators, such as inhibitors of receptor-interacting serine/threonine-protein kinase 1 (RIPK1), ferroptosis modulators, and autophagy blockers, are beginning to show promise in preclinical rheumatic disease models, opening potential translational pathways from transcriptomic findings to therapeutic innovations (42–44). In particular, NAMPT is a druggable enzyme with existing inhibitors explored in other inflammatory conditions (45) while DDIT3 and its upstream ER−stress pathway represent emerging therapeutic targets (46). Although GATA2 deficiency is classically associated with immunodeficiency and myeloid neoplasia, recent studies highlight its role as a master regulator of endothelial function through miRNA networks such as miR−126 and miR−221, which are critical for vascular homeostasis and angiogenesis. Thus, GATA2 downregulation in AS may contribute to vascular inflammation or impaired endothelial repair —processes relevant to AS-related cardiovascular comorbidities—and could represent a novel biomarker or therapeutic target for modulating immune−vascular crosstalk in this disease (47). The convergent dysregulation of these genes across multiple regulatory layers nominates them as high−priority candidates for further validation as potential biomarkers or therapeutic targets in AS.

Despite these advances, this study has limitations. The relatively small sample size limits statistical power and may introduce cohort-specific bias. Since HLA-B27 itself could drive broad transcriptional and splicing changes in blood, without including HLA-B27 positive healthy controls in this study, we cannot exclude the possibility that some of the disease-related changes in transcript expression and alternative splicing are resulted from HLA-B27. Although our analysis revealed compelling transcript-level signatures, it remains descriptive at the transcriptomic level. Functional validation through protein-level assays, isoform−specific manipulations in immune cells, and investigations in disease−relevant tissues are essential next steps to establish causality and clarify the physiological impact of the identified isoform switches and splicing events. Isoform-specific knockdown or overexpression experiments in immune cells or fibroblasts could help clarify causal relationships.

## Conclusion

In conclusion, our long-read transcriptomic profiling of AS provides novel insights into the multilayered regulation of gene activity, especially as it pertains to PCD-related pathways. Beyond revealing differential gene and transcript expression, the study uniquely captures isoform-specific switching and splicing changes that reshape immune signaling architecture in AS. Among the key findings, *NAMPT*, *GATA2*, and *DDIT3* emerged as candidate regulators situated at the intersection of inflammation and cell death. While the exploratory cohort size warrants caution in over-interpretation, the integration of multi-layer analyses, external dataset validation, and the generation of specific, testable hypotheses substantially strengthen the biological plausibility of these findings. These results not only enhance our mechanistic understanding of AS pathogenesis but also nominate high-priority targets for future validation and therapeutic exploration.

## Data Availability

The datasets generated and/or analysed during the current study are available in Gene Expression Omnibus (GEO) repository (https://www.ncbi.nlm.nih.gov/geo/query/acc.cgi?acc=GSE299639).

## Supplementary material

Supplementary Figure 1 Schematic diagram of the experimental and data-analysis procedures in this study.

Supplementary Figure 2 Long-read sequencing reveals transcript expression. **(A)** Schematic diagram of the experimental and data-analysis procedures in this study. **(B)** Transcript classification codes based on their relationship to reference transcripts, as generated by GffCompare. **(C)** The bar plots showing the classification of transcripts with quantification. **(C, D)** The top 10 most enriched GO terms (biological process) and KEGG pathway were illustrated for genes of new transcript.
