# Supplementary figures and images for: Integrated long-read transcriptomic profiling of peripheral blood from ankylosing spondylitis patients identifies regulatory shifts and core genes associated with programmed cell death
Source: Front Immunol. 2026 Mar 6;17:1640271. doi: 10.3389/fimmu.2026.1640271 (PMC13002829; doi:10.3389/fimmu.2026.1640271)

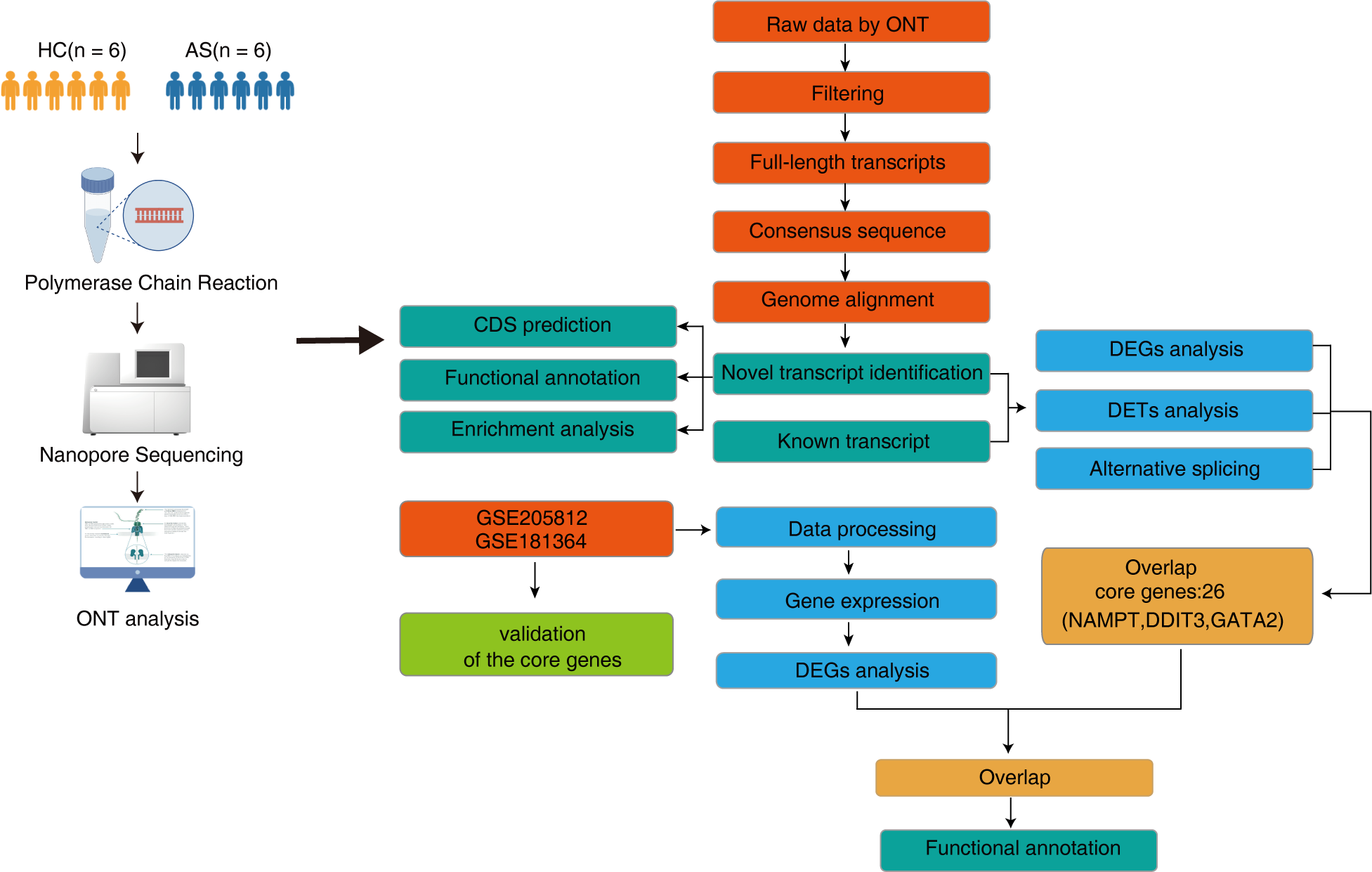

Supplement: Supplementary Figure 1 — Schematic diagram of the experimental and data-analysis procedures in this study. [file Image1.tif]

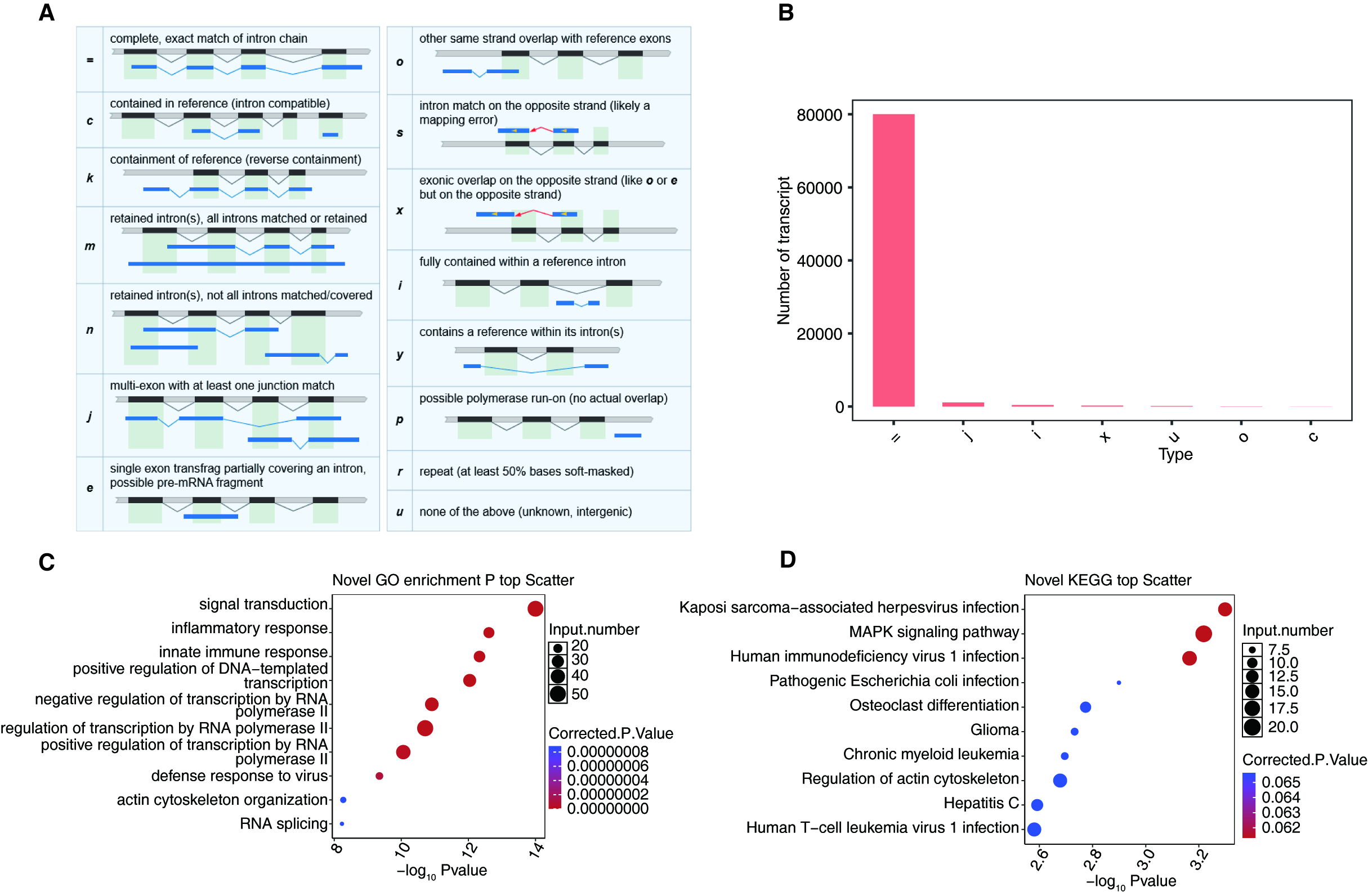

Supplement: Supplementary Figure 2 — Long-read sequencing reveals transcript expression. (A) Schematic diagram of the experimental and data-analysis procedures in this study. (B) Transcript classification codes based on their relationship to reference transcripts, as generated by GffCompare. (C) The bar plots showing the classification of transcripts with quantification. (C, D) The top 10 most enriched GO terms (biological process) and KEGG pathway were illustrated for genes of new transcript. [file Image2.tif]
